# Hyperoside Promotes Mitochondrial Autophagy Through the miR-361-5p/PI3K/Akt/mTOR Signaling Pathway, Thereby Improving UVB-Induced Photoaging

**DOI:** 10.3390/antiox14121401

**Published:** 2025-11-25

**Authors:** Danni Yang, Jiayi Le, Shuyun Xiao, Yulin Cui, Wanfang Zhu, Kouharu Otsuki, Wei Li, Jian Xu, Feng Feng, Jie Zhang

**Affiliations:** 1 School of Traditional Chinese Pharmacy, China Pharmaceutical University, Nanjing 210009, China; 3323020975@stu.cpu.edu.cn (D.Y.);; 2 College of Pharmacy, Changchun University of Chinese Medicine, Changchun 130022, China; 3 Faculty of Pharmaceutical Sciences, Toho University, Miyama 2-2-1, Funabashi 274-8510, Chiba, Japanliwei@phar.toho-u.ac.jp (W.L.)

**Keywords:** photoaging, hyperoside, miRNA, PI3K/Akt/mTOR, mitophagy

## Abstract

Ultraviolet radiation B (UVB) radiation can induce oxidative stress, DNA damage, and inflammation, leading to skin wrinkling, impaired barrier function, and an increased risk of cancer. Addressing or preventing photoaging may provide a promising therapeutic avenue for these conditions. Hyperoside (HY), a compound abundantly found in medicinal plants including *Hypericum perforatum* and *Crataegus*, has been reported to have various pharmacological activities such as antioxidant, anti-inflammatory, cytoprotective, and antitumor effects; however, there are currently no studies systematically exploring the potential and mechanisms of HY in alleviating skin damage caused by ultraviolet (UV) rays. We investigated the inhibitory effects of HY on oxidative stress responses, reducing keratinocyte aging. HY can also exert these effects by mediating the PI3K/AKT/mTOR signaling pathway through miR-361-5p, maintaining mitochondrial dynamic stability, alleviating mitochondrial dysfunction, and enhancing mitophagy. Additionally, in vivo, HY was able to significantly improve skin wrinkles in mice while reducing changes in thickness and aging of the epidermis and dermis.

## 1. Introduction

Prolonged exposure to sunlight is a well-established cause of photoaging, also referred to as extrinsic aging. This process is characterized by degradation of dermal collagen, alterations in elastic fiber architecture, and increased mutagenesis within melanocytes [1,2]. Solar ultraviolet radiation (UVR) induces cellular senescence in skin cells, leading to the secretion of senescence-associated secretory phenotype (SASP) factors by keratinocytes. Concurrently, it causes intracellular accumulation of excess reactive oxygen species (ROS) and impairs mitochondrial function [3]. As the primary site of aerobic respiration, mitochondria play a pivotal role in the progression of cellular senescence [4,5]. UVB (280–320 nm) represents the most harmful segment of the ultraviolet spectrum and plays a central role in triggering skin photoaging and DNA damage. Furthermore, substantial evidence indicates that excessive UVB exposure may even contribute to the development of skin malignancies, including melanoma [1,6]. Although various interventions—such as the use of sunscreen, laser therapy, and radiofrequency treatments—have been employed to mitigate photoaging, their long-term efficacy remains limited [7]. Consequently, the development of safe and effective anti-photoaging pharmaceuticals represents an urgent research priority.

MicroRNAs (miRNAs or miRs) are short (~22 nucleotides), single-stranded non-coding RNA molecules that function as highly conserved post-transcriptional regulators of gene expression. Although miRNAs do not encode proteins, they modulate the expression of protein-coding genes by binding to target mRNAs [8]. For instance, Emad et al. (2023) reported that miR-361-5p exhibits an antagonistic role in cellular senescence and resveratrol-induced regeneration, suggesting its potential as a therapeutic target in aging interventions [9]. Importantly, this anti-senescence function appears to be universal. Further evidence indicates that dysregulation of miR-361-5p is associated with various stress responses, and one of its recognized molecular functions is the regulation of core cell signaling pathways. Studies have demonstrated that miR-361-5p plays a role in cutaneous squamous cell carcinoma by targeting vascular endothelial growth factor A (VEGFA), whereby suppression of this miRNA can inhibit the malignant behaviors of the cancer cells [10]. Furthermore, the vascular endothelial growth factor/vascular endothelial growth factor receptor (VEGF/VEGFR) signaling axis regulates the growth of melanoma cells through activation of the downstream phosphoinositide 3-kinase/protein kinase B (PI3K/AKT) pathway. Melanoma is the primary cause of most skin cancer-related deaths [11]. In another study, Tian et al. demonstrated that miR-361-5p attenuates chemotherapy resistance in gastric cancer cells by targeting forkhead box protein M1 (FOXM1) and activating the PI3K/Akt signaling pathway [12]. Furthermore, preliminary bioinformatic analysis (e.g., using TargetScan (version 3.1)) predicts that PI3K subunits are potential direct targets of miR-361-5p. This comprehensive evidence suggests that miR-361-5p may function as an upstream regulator of the PI3K/Akt pathway, whose regulatory mechanisms in skin photoaging remain to be explored. The PI3K/Akt pathway is critically involved in regulating essential cellular processes, including survival, proliferation, metabolism, and cytoskeletal reorganization. Notably, upon UVB irradiation, PI3K signaling activation induces transcription of matrix metalloproteinase-1 (MMP-1), thereby promoting cellular senescence [13]. Natural products have garnered increasing attention in recent years for their multi-target capacity to ameliorate UVB-induced skin aging [14]. Hyperoside (HY), a flavonol glycoside found in plants of the families *Hypericaceae*, *Campanulaceae*, *Rosaceae*, *Fabaceae*, and *Ericaceae*, has been reported to exhibit diverse pharmacological activities, including antioxidant, anti-inflammatory, cytoprotective, and anti-tumor effects [15,16]. However, no study to date has comprehensively investigated the potential of HY in alleviating UVB-induced skin damage.

Notably, existing studies suggest a potential functional connection between HY, miR-361-5p, and the PI3K/Akt pathway. For instance, HY has been reported to modulate autophagy via the PI3K/Akt pathway in protecting trastuzumab-induced cardio-toxicity [17]. Concurrently, as mentioned earlier, miR-361-5p has also been reported to target and influence PI3K/Akt signaling [12]. Given that the PI3K/Akt/mammalian target of rapamycin (mTOR) pathway is a master regulator of mitochondrial function and cellular senescence, we hypothesize that HY might mitigate UVB-induced mitochondrial dysfunction and cellular senescence by modulating miR-361-5p to influence this pathway.

## 2. Materials and Methods

### 2.1. Cell Lines, Compounds, Reagents, and Laboratory Instruments

The human keratinocyte cell line HaCaT (SNL-163) was obtained from Wuhan SUNNCELL (Wuhan, China). Hyperoside (HY; purity ≥ 98%, CAS No.: 482-36-0) was purchased from Beijing Solarbio Science and Technology Co., Ltd. (Jiangsu, China). The following assay kits were acquired from Beyotime Biotechnology (Shanghai, China): BCA Protein Concentration Assay Kit (P0012), Methylthiazolyldiphenyl-tetrazolium Bromide (MTT) Cell Viability Kit (ST316), Autophagy Staining Kit (C3018S), Senescence-Associated β-galactosidase Staining Kit (C0602), Reactive Oxygen Species Assay Kit (CA1410), and EdU-555 Cell Proliferation Assay Kit (C0075S). The Cell Cycle Detection Kit (KGA511) was supplied by KeyGEN BioTECH (Nanjing, China). MitoSOX Red Mitochondrial Superoxide Indicator (HY-D1055) was procured from MedChemExpress (Shanghai, China).

Antibodies were sourced as follows: p21 (10355-1-AP), Beclin1 (11306-1-AP), GAPDH (60004-1-Ig), LC3 (14600-1-AP), PI3K (20584-1-AP), p-Akt (66444-1-Ig), LaminB1 (12987-1-AP), TOM20 (11802-1-AP), and Tubulin (11224-1-AP) were purchased from Proteintech Group (Wuhan, China); γ-H2AX (M63324S) was from Abmart (Shanghai, China); Akt (YP-mAb-14652), mTOR (YP-mAb-14867), p-mTOR (YP-mAb-14329), and p-PI3K (YP-Ab-17956) were obtained from Upingbio (Hangzhou, China); p16 (CY5316), PINK1 (BY0130), Parkin (CY6641), and p62 (CY9081) were from Abways Technology (Shanghai, China).

### 2.2. Cell Culture

HaCaT cells were cultured in high-glucose Dulbecco’s Modified Eagle Medium (DMEM) supplemented with 10% fetal bovine serum at 37 °C in a 5% CO_2_ humidified atmosphere. Upon reaching 90% confluence, cells were detached using trypsin and subcultured. All experiments were conducted using cells between passages 2 and 7 during the exponential growth phase.

### 2.3. Methylthiazolyldiphenyl-Tetrazolium Bromide (MTT) Assay

HaCaT cells were seeded into 96-well plates at a density of 1 × 10^4^ cells per well and treated with various concentrations of HY (1–1000 μM) for 24 h. For UVB irradiation experiments, a UVB lamp (280–315 nm spectrum) was positioned 5 cm above the culture dish, and cells were exposed to doses ranging from 20 to 140 mJ/cm^2^. After irradiation, cells were incubated for an additional 24 h. Subsequently, 10 μL of MTT solution (5 mg/mL) was added to each well, and the plate was incubated for 4 h. The medium was then aspirated, and 150 μL of dimethyl sulfoxide (DMSO) was added to dissolve the formazan crystals. Absorbance was measured at 490 nm using a microplate reader (Molecular Devices, LLC, San Jose, CA, USA).

### 2.4. Functional Assays

To evaluate the protective effects of HY against UVB-induced damage, a series of functional assays was performed. Unless otherwise specified, cells were pretreated with the indicated concentrations of HY for 24 h, exposed to a specific dose of UVB (detailed in each assay below), and then further incubated for subsequent analysis. All experiments were independently repeated in triplicate.

#### 2.4.1. Cell Migration Assay

Cell migration was assessed using 24-well Transwell chambers (Corning Incorporated, Corning, NY, USA). HaCaT cells were resuspended in serum-free medium and seeded into the upper chamber at a density of 5 × 10^4^ cells per chamber. Medium containing 10% fetal bovine serum (FBS) was added to the lower chamber as a chemoattractant. UVB irradiation (60 mJ/cm^2^) was applied immediately after seeding. After incubation for 24 h at 37 °C, non-migrated cells on the upper surface of the membrane were removed with a cotton swab. Cells that migrated to the lower surface were fixed with 4% paraformaldehyde and stained with 0.1% crystal violet. The stained cells were counted from three randomly selected fields under an optical microscope.

#### 2.4.2. Clonogenic Survival Assay

HaCaT cells were seeded at a low density (500 cells per dish) into 6 cm culture dishes. After attachment, cells were exposed to UVB (60 mJ/cm^2^) and immediately replenished with fresh medium containing indicated concentrations of HY. Cells were cultured for 10 days at 37 °C with 5% CO_2_, with the medium changed every 3 days. After cultivation, cells were gently washed with PBS, fixed with 4% paraformaldehyde, and stained with 0.1% crystal violet.

#### 2.4.3. Senescence-Associated β-Galactosidase (SA-β-gal) Staining

Cells were seeded into 6-well plates at a density of 2 × 10^5^ cells per well. After UVB irradiation (60 mJ/cm^2^) and HY treatment, cells were further cultured for 72 h. Subsequently, cells were stained using a commercial SA-β-gal staining kit (KeyGEN BioTECH, Nanjing, China) strictly following the manufacturer’s protocol. The percentage of SA-β-gal-positive (blue) cells was observed and counted under an optical microscope.

#### 2.4.4. 5-Ethynyl-2′-Deoxyuridine (EdU) Proliferation Assay

Cell proliferation was detected using the Cell-Light EdU Apollo 567 In Vitro Imaging Kit (KeyGEN BioTECH, Nanjing, China). Cells were seeded in 96-well plates (1 × 10^4^ cells/well). After treatments, they were incubated with 10 μM EdU for 2 h at 37 °C. Cells were then fixed, permeabilized, and subjected to a click reaction to label the incorporated EdU according to the manufacturer’s instructions. Nuclei were counter-stained with Hoechst 33342. The ratio of EdU-positive cells (red) to total cells (blue) was observed and calculated using a fluorescence microscope (Nikon Instruments Inc., Tokyo, Japan).

#### 2.4.5. Cell Cycle Analysis

The cell cycle was analyzed by flow cytometry. After treatments, cells (including floating cells in the supernatant) were collected, washed with ice-cold PBS, and fixed in 70% ethanol overnight at −20 °C. After fixation, cells were washed with PBS and stained with a propidium iodide (PI, 50 μg/mL) solution containing 0.1 mg/mL RNase A in the dark at 37 °C for 30 min. DNA content was detected using a BD Accuri C6 flow cytometer (Becton, Dickinson and Company, Franklin Lakes, NJ, USA), and cell cycle distribution (G0/G1, S, and G2/M phases) was analyzed using ModFit LT (or FlowJo) software (version 10.8.1) [18,19,20].

### 2.5. Quantitative Real-Time PCR (RT-qPCR)

Total RNA was extracted from HaCaT cells using TRIzol reagent (Invitrogen, Carlsbad, CA, USA) according to the manufacturer’s instructions. cDNA was synthesized from 1000 ng of RNA using a reverse transcription kit (Takara Bio Inc., Kusatsu, Shiga, Japan). Quantitative PCR was performed using SYBR^®^ Premix Ex Taq™ II (Takara Bio Inc., Kusatsu, Shiga, Japan) in a 20 μL reaction mixture containing forward and reverse primers and cDNA template. Amplification was carried out for 40 cycles under the following conditions: initial denaturation at 95 °C for 30 s; amplification at 95 °C for 5 s and 60 °C for 1 min; melting curve analysis at 95 °C for 1 s, 55 °C for 55 s, and 97 °C for 1 s; and final cooling at 37 °C for 30 s. The LightCycler^®^ 96 System was used for detection. GAPDH served as the internal control. Primer sequences are listed in Table 1.

### 2.6. Western Blot Analysis

Proteins were extracted from skin tissues or HaCaT cells using high-efficiency RIPA lysis buffer according to the manufacturer’s protocol. Protein concentration was quantified using a bicinchoninic acid (BCA) assay kit (KeyGEN BioTECH, Nanjing, China). Samples (4 μg/μL, 6 μL per lane) were separated by 10% sodium dodecyl sulfate—polyacrylamide gel electrophoresis (SDS-PAGE) and transferred onto polyvinylidene fluoride (PVDF) membranes (Merck Millipore, Burlington, MA, USA). Membranes were blocked with 5% skim milk for 2.5 h, incubated with primary antibodies at 4 °C overnight, and then probed with appropriate secondary antibodies for 2 h at room temperature. Protein bands were visualized using an automated chemiluminescence imaging system and analyzed with ImageJ software (v1.8.0).

### 2.7. Immunofluorescence

HaCaT cells were cultured on confocal dishes and treated as indicated. After treatment, cells were fixed with 4% formaldehyde for 20 min at 4 °C, washed three times with PBS, and permeabilized with 0.2% Triton X-100 for 30 min. Blocking was performed with 10% goat serum for 30 min at room temperature. Cells were incubated with primary antibodies overnight at 4 °C, followed by incubation with fluorescently labeled secondary antibodies for 1.5 h in the dark. The primary antibodies and dilutions used were as follows: anti-γ-H2AX antibody at 1:500; anti-LaminB1 antibody at 1:500; anti-Tubulin antibody at 1:1000; anti-TOM20 antibody at 1:200; anti-Beclin1 antibody at 1:300; anti-LC3B anti-body at 1:200; anti-p21 antibody at 1:500. All images were acquired using a Nikon A1R+ confocal microscope system with consistent acquisition parameters to ensure comparability [21].

### 2.8. Detection of ROS and Mitochondrial Superoxide

HaCaT cells were seeded in 6-well plates at 1 × 10^5^ cells per well and pretreated with HY (1 or 5 μm) for 24 h prior to UVB irradiation. After UVB exposure, cells were incubated with 10 μm 2′,7′-dichlorofluorescin diacetate (DCFH-DA) or 5 μm MitoSOX reagent for 30 min at 37 °C in the dark. Following three washes with PBS, fluorescence was quantified by flow cytometry or visualized under a fluorescence microscope.

### 2.9. Transmission Electron Microscopy

Samples were high-pressure frozen using a Leica EMPACT2 system and subjected to freeze substitution in anhydrous acetone containing 1% OsO_4_ and 0.1% uranyl acetate using a Leica EM AFS2 unit (Leica Microsystems, Wetzlar, Germany). The temperature was raised stepwise from −85 °C to 0 °C over several days. Samples were embedded in Spurr resin, and ultrathin sections were cut with a Leica EM UC6 ultramicrotome (Leica Microsystems, Wetzlar, Germany). Sections were stained with uranyl acetate and lead citrate and examined under a Philips CM100 transmission electron microscope operating at 100 kV.

### 2.10. Network Pharmacology Analysis

The 2D structure of HY was obtained from the PubChem database (https://pubchem.ncbi.nlm.nih.gov/ accessed on 13 May 2025, Compound CID: 5,320,951). Potential protein targets of HY were predicted using the following online platforms: SwissTargetPrediction (http://www.swisstargetprediction.ch/ accessed on 13 May 2025) with the species set to “Homo sapiens”; and PharmMapper (http://www.lilab-ecust.cn/pharmmapper/ accessed on 13 May 2025) with the species set to “Human”. All predicted targets were merged, and duplicates were removed to obtain the potential target set of HY. Targets related to “photoaging” were retrieved by searching the following databases: DisGeNET (https://www.disgenet.org/, version 7.0 accessed on 23 May 2025), GeneCards (https://www.genecards.org/, version 5.14, accessed on 23 May 2025), OMIM (https://www.omim.org/ accessed on 23 May 2025), and DrugBank (https://go.drugbank.com/, version 5.1.9 accessed on 23 May 2025). All database searches were conducted between 13 and 23 May 2025. Using keywords such as “photoaging”, “skin aging”, and “UV-induced skin aging”, results from each database were combined and deduplicated to generate the disease target set. Common targets between HY and photoaging were identified by intersecting the respective target sets. A protein–protein interaction (PPI) network was constructed using the STRING database (https://string-db.org/, version 12.0 accessed on 27 May 2025). The key parameters were set as follows: organism limited to “Homo sapiens”; a minimum required interaction score of >0.90 (highest confidence); and disconnected nodes were hidden in the network. The resulting TSV data were imported into Cytoscape software (version 3.10.3) for network visualization and topological analysis. The CytoNCA plugin was used to identify hub targets in the network based on parameters including “Degree Centrality”, “Betweenness Centrality”, and “Closeness Centrality”. The crystal structures of the core targets PI3K (PDB ID: 5JHB) or Akt (PDB ID: 1H10) were downloaded from the PDB database (https://www.rcsb.org/ accessed on 29 May 2025). Pre-docking preparations were performed using AutoDock Tools 1.5.7: water molecules and the original ligand were removed, polar hydrogen atoms were added, and Gasteiger charges were calculated. The 3D structure of HY was energy-minimized and set as a flexible ligand. The docking grid box was centered on and covered the active site of PI3K. Molecular docking was performed using AutoDock Vina 1.1.2, with the exhaustiveness parameter set to 32. The resulting binding conformations were ranked by binding affinity (unit: kcal/mol), and the binding modes were visualized and analyzed using PyMOL (version 2.5.0).

### 2.11. Transfection

MiR-361-5p mimic and si-PI3K, along with their respective negative controls, were purchased from GenePharma (Shanghai, China). HaCaT cells in the logarithmic growth phase were seeded into 6-well plates at 5 × 10^5^ cells per well and transfected using GP-transfect-mate reagent (Genepharma Co., Ltd., Shanghai, China) according to the manufacturer’s instructions.

### 2.12. Animal Experiments

Forty-eight male Institute of Cancer Research (ICR) mice (4–5 weeks old, 17 ± 1 g) were supplied by Jiangsu Qinglongshan Biotechnology Co., Ltd (Nanjing, China). Mice were housed under a 12 h light/dark cycle with ad libitum access to food and water. All procedures were approved by the Institutional Animal Care and Use Committee of China Pharmaceutical University (Approval No.: 2025-01-001. Animal Approval Date: 1 January 2025) and complied with the Animal Research: Reporting of In Vivo Experiments (ARRIVE) guidelines. Mice were randomly divided into six groups (*n* = 8 per group): control, UVB model, low-dose HY (LH) (10 mg/mL), medium-dose HY (MH) (20 mg/mL), high-dose HY (HH) (40 mg/mL), and vitamin E (VE) positive control. Dorsal hair was removed prior to UVB irradiation. Mice were irradiated with 150 mJ/cm^2^ UVB three times per week. Topical applications of HY or VE at the indicated concentrations were administered 30 min before each irradiation. The solutions were applied evenly to the shaved dorsal skin area (approximately 4 cm × 5 cm). A fixed volume of 100 μL was applied per mouse, delivering doses of 10, 20, and 40 mg/kg for the low-, medium-, and high-dose HY groups, respectively (based on an average mouse weight of 25 g, this equates to 0.5, 1.0, and 2.0 mg of HY per cm^2^ of skin). At the endpoint, dorsal skin samples were collected for analysis [22].

### 2.13. Histopathological Analysis

Skin tissues were fixed, paraffin-embedded, sectioned, and dewaxed. Masson’s trichrome staining was used to evaluate collagen content, and hematoxylin and eosin (H&E) staining was applied to assess histological changes. Slides were scanned using a NanoZoomer 2.0 RS digital slide scanner and analyzed with ImageJ.

### 2.14. Immunohistochemistry (IHC)

Paraffin sections were dewaxed, rehydrated, and subjected to antigen retrieval. Endogenous peroxidase activity was blocked, and sections were incubated with primary and secondary antibodies. Signal was developed using 3,3-*N*-diaminobenzidine tertrahydrochloride (DAB), and nuclei were counterstained with hematoxylin. Slides were dehydrated, mounted, and imaged under a microscope, and analyzed with ImageJ software (version 1.54f) [23].

### 2.15. Statistical Analysis

Data were analyzed using GraphPad Prism 10 and expressed as mean ± standard error of the mean (SEM). For normally distributed data with homogeneous variance, one-way analysis of variance (ANOVA) was applied; otherwise, non-parametric tests were used. A *p*-value < 0.05 was considered statistically significant.

## 3. Results

### 3.1. Protective Effect of HY Against UVB-Induced Skin Damage In Vitro

**Figure 1:**
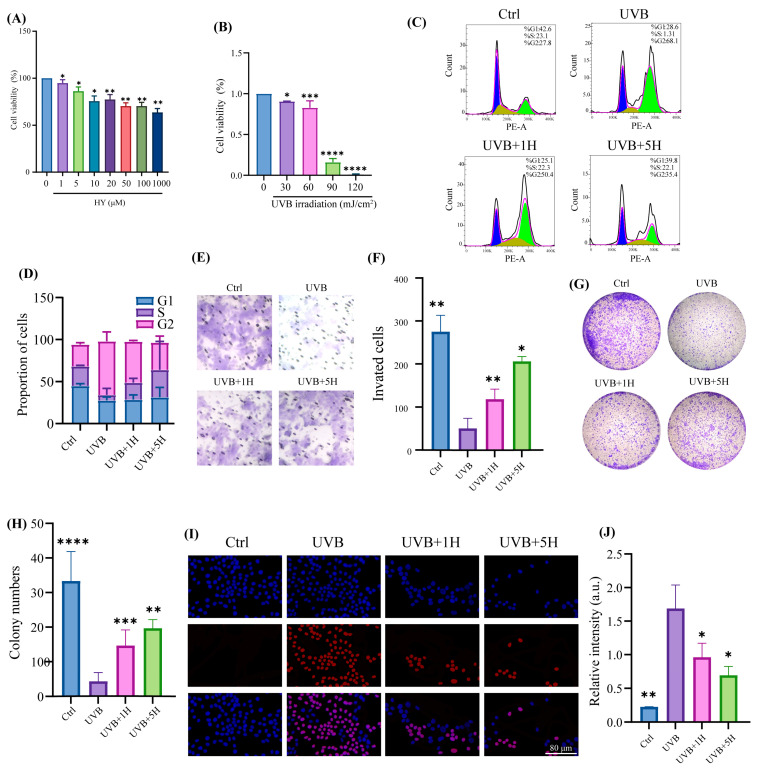
HY protected HaCaT from UVB-induced cell damage. (**A**) Cytotoxicity of HY to HaCaT cells. (**B**) Cytotoxicity of different UVB powers to HaCaT cells. (**C**) The regulatory effect of HY on the cell cycle. (**D**) Proportion of cells at different periods. (**E**,**F**) Transwell assay and statistical analysis of cell invasion and migration counts. (**G**,**H**) Detecting the colony formation of HaCaT cells. (**I**,**J**) HaCaT treated with HY was measured using an EDU assay, and the number of EDU-positive cells (Red) (Scale bars: 80 μm). Data are presented as the mean ± SD (n = 3). * *p* < 0.05, ** *p* < 0.01, *** *p* < 0.001 and **** *p* < 0.0001. Ctrl (control group), UVB (the irradiated group), UVB+1H (1 μm of HY), UVB+5H (5 μm of HY).

Based on the pharmacological profile of HY, we hypothesized that it might exert protective effects against UVB radiation-induced skin aging. To test this hypothesis, we first evaluated the impact of varying concentrations of HY on the viability of HaCaT cells. Results indicated that HY at concentrations of 1 μm and 5 μm did not exhibit significant cytotoxicity (Figure 1A). Using the MTT assay, we assessed the influence of different UVB irradiation intensities on cell viability and established an irradiation dose of 60 mJ/cm^2^ as a standardized parameter for subsequent experiments (Figure 1B). Cell cycle analysis revealed that UVB irradiation induced G2/M phase arrest, which was effectively reversed by HY treatment, with the most pronounced effect observed at 5 μm (Figure 1C,D). Furthermore, cell invasion assays demonstrated that HY significantly alleviated UVB-induced suppression of HaCaT cell migration (Figure 1E,F). Colony formation assays indicated that UVB exposure markedly reduced cell viability, whereas HY treatment notably enhanced proliferative capacity (Figure 1G,H), consistent with previously reported findings [21]. Immunofluorescence staining showed a substantial increase in apoptosis in UVB-irradiated cells, which was markedly attenuated by HY treatment (Figure 1I,J). In summary, HY effectively counteracts UVB-induced reduction in HaCaT cell viability and suppresses apoptosis, demonstrating significant protective activity.

### 3.2. HY Protects HaCaT from UVB-Induced Cellular Senescence

**Figure 2:**
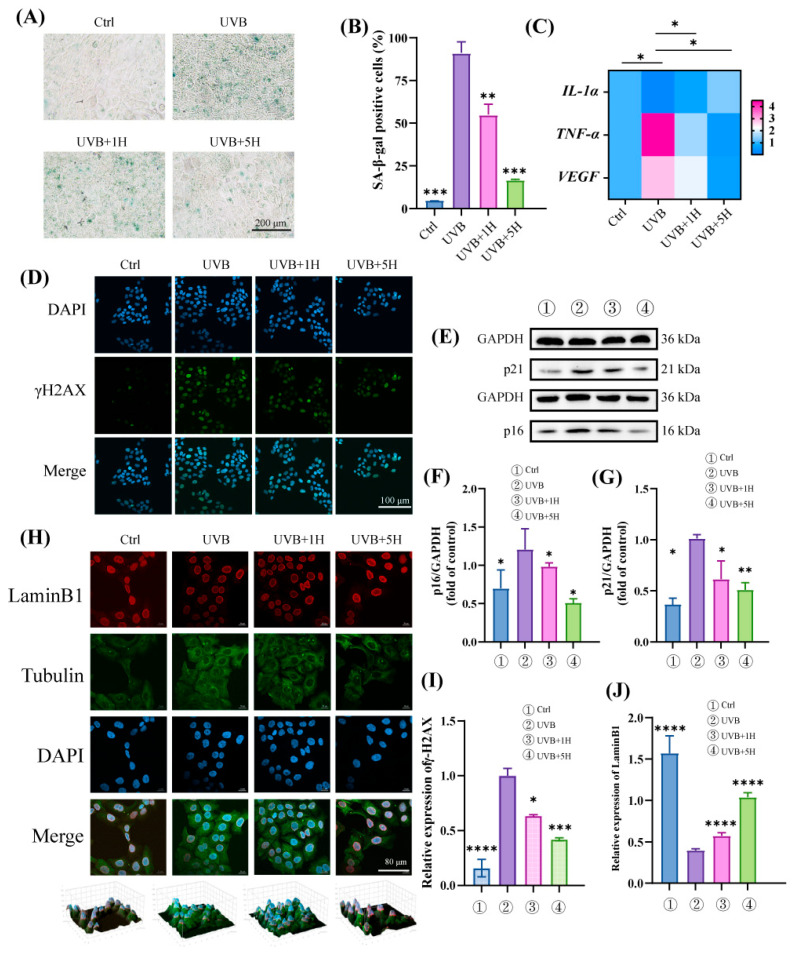
HY protects HaCaT from UVB-induced cellular senescence. (**A**,**B**) Representatives of SA-β-gal staining imaging and quantitative analysis. (**C**) The mRNA expressions of *IL-1α*, *TNF-α*, and *VEGF* were determined by RT-qPCR; (**D**) Representative images of γH2AX staining (Scale bars: 100 μm). The cells were immunostained with γH2AX (green) and Nuclei were stained with DAPI (blue). (**E**) Western blot detected aging markers p21 and p16. (**F**,**G**) quantitative analysis of p21 and p16. (**H**) Immunofluorescence assay for the expression of LaminB1 (Scale bars: 80 μm). The cells were immunostained with Tubulin (green), LaminB1 (red) and Nuclei were stained with DAPI (blue). (**I**) Expression of γH2AX fluorescence intensity. (**J**) Relative fluorescence intensity of LaminB1. Data are presented as the mean ± SD (n = 3). * *p* < 0.05, ** *p* < 0.01, *** *p* < 0.001 and **** *p* < 0.0001. Ctrl (control group), UVB (the irradiated group), UVB 1H (1 μm of HY), UVB 5H (5 μm of HY).

To evaluate the effect of HY on UVB-induced cellular senescence, we performed SA-β-gal staining on HaCaT cells. The results indicated that the number of SA-β-gal-positive cells increased significantly after UVB irradiation, whereas HY treatment markedly reduced the proportion of positive cells (Figure 2A,B). Cellular senescence is often accompanied by the secretion of the senescence-associated secretory phenotype (SASP) [24], which comprises various pro-inflammatory cytokines, growth factors, chemokines, and matrix-remodeling enzymes [25]. Accordingly, we examined the mRNA expression levels of *IL-1α, TNF-α,* and *VEGF*. HY significantly suppressed the expression of *TNF-α* and *VEGF* while upregulating *IL-1α* (Figure 2C), with the most pronounced effects observed at a concentration of 5 μm. Furthermore, p16 and p21 are well-established and reliable markers of senescence [26]. As shown in Figure 2E–G, UVB irradiation notably increased the expression of p16 and p21, and this effect was reversed by HY treatment in a concentration-dependent manner. The phosphorylated form of histone H2AX (γH2AX) serves as a marker of the DNA damage response (DDR) [27]. Its phosphorylation level was significantly reduced following HY treatment (Figure 2D,I). Senescent cells often undergo morphological alterations accompanied by downregulation of LaminB1, establishing LaminB1 as an indicator of senescence [28]. Confocal microscopy revealed that the fluorescence intensity of LaminB1 was significantly weakened in the UVB group, whereas high concentrations of HY noticeably enhanced its expression (Figure 2H,J). These results demonstrate that HY effectively protects HaCaT cells from UVB-induced cellular senescence.

### 3.3. HY Induce Autophagy and Attenuate Mitochondrial Damage

**Figure 3:**
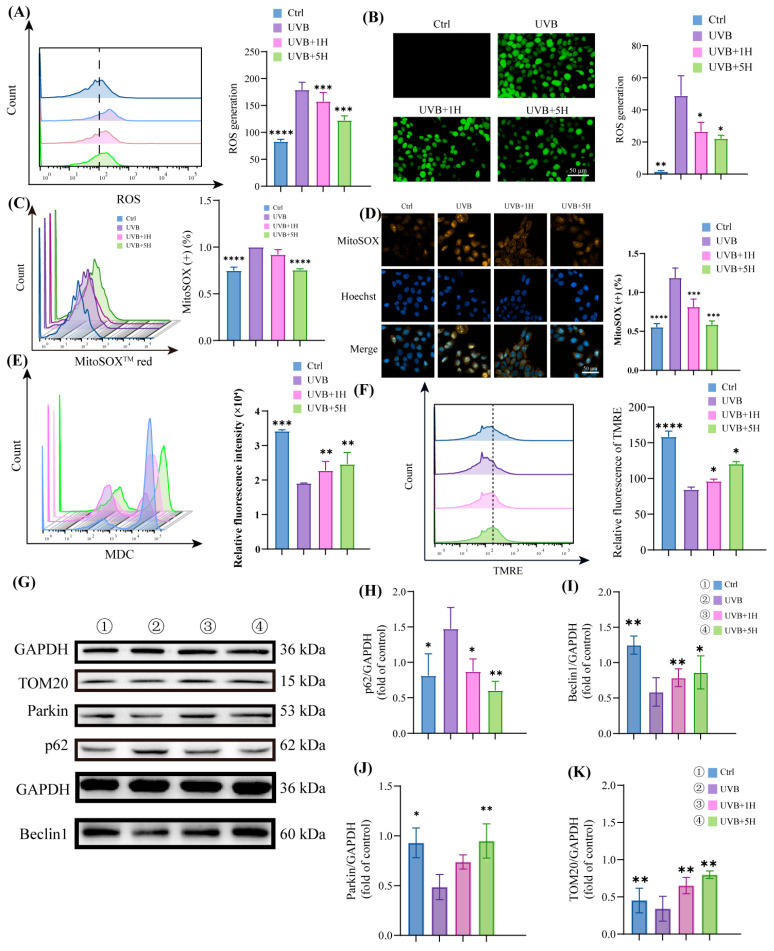
HY stimulates autophagy and attenuates mitochondrial damage. (**A**,**B**) Representative images and flow cytometry analysis of ROS (green) (Scale bars: 50 μm). (**C**,**D**) Representative images and flow cytometry analysis of the intramitochondrial superoxide MitoSOX (Scale bars: 50 μm). The cells were immunostained with MitoSOX (yellow) and Nuclei were stained with DAPI (blue). (**E**) Flow cytometry for the detection of MDC. (**F**) Flow cytometry for TMRE. (**G**–**K**) Western blot detected the signature proteins p62 and Beclin1, as well as mitochondrial autophagy-related proteins TOM20 and Parkin, and quantitative analysis was performed. Data are presented as the mean ± SD (n = 3). * *p* < 0.05, ** *p* < 0.01, *** *p* < 0.001 and **** *p* < 0.0001. Ctrl (control group), UVB (the irradiated group), UVB 1H (1 μm of HY), UVB 5H (5 μm of HY).

ROS have been demonstrated to play a critical role in skin photoaging [29]. Using flow cytometry and immunofluorescence assays, we observed a significant increase in DCFH-DA-positive cells following UVB irradiation, whereas HY treatment markedly reduced intracellular ROS levels (Figure 3A,B). Mitochondria represent a major target of ROS, and severe oxidative damage often leads to mitochondrial dysfunction [30]. Further analysis via flow cytometry and immunofluorescence confirmed that UVB irradiation elevated mitochondrial superoxide (MitoSOX) production in HaCaT cells, with the mean fluorescence intensity increasing approximately 1.4-fold compared to the control group. HY treatment effectively suppressed this UVB-induced rise in MitoSOX (Figure 3C,D).

Additionally, monodansylcadaverine (MDC), a fluorescent dye commonly used to label autophagic vesicle structures, was employed to evaluate the formation of autophagic vesicles. The results revealed that HY treatment significantly increased the number of MDC-labeled vesicles in HaCaT cells—approximately 1.8-fold higher than that in the UVB group (Figure 3E). Meanwhile, TMRE staining was used to assess mitochondrial membrane potential as an indicator of membrane integrity. UVB irradiation resulted in a pronounced loss of mitochondrial membrane potential, which was notably reversed by HY treatment (Figure 3F).

Western blot analysis indicated altered autophagic processes in UVB-exposed cells, characterized by decreased p62 degradation and downregulated Beclin1 expression. In contrast, HY treatment led to a significant reduction in p62 and upregulation of Beclin1. Notably, HY administration also increased the protein levels of Parkin and TOM20. The increase in Parkin is consistent with early events in the initiation of mitophagy. However, the concurrent increase in TOM20, a mitochondrial outer membrane protein, is incongruent with its typical degradation during the completion of mitophagy. This pattern may suggest a complex dynamic balance between mitophagic initiation and mitochondrial biogenesis under HY intervention, or a potential blockage in the later stages of the mitophagic flux. Further investigation is required to elucidate the precise mechanism underlying this observation (Figure 3G–K).

### 3.4. HY Promotes Mitophagy

**Figure 4:**
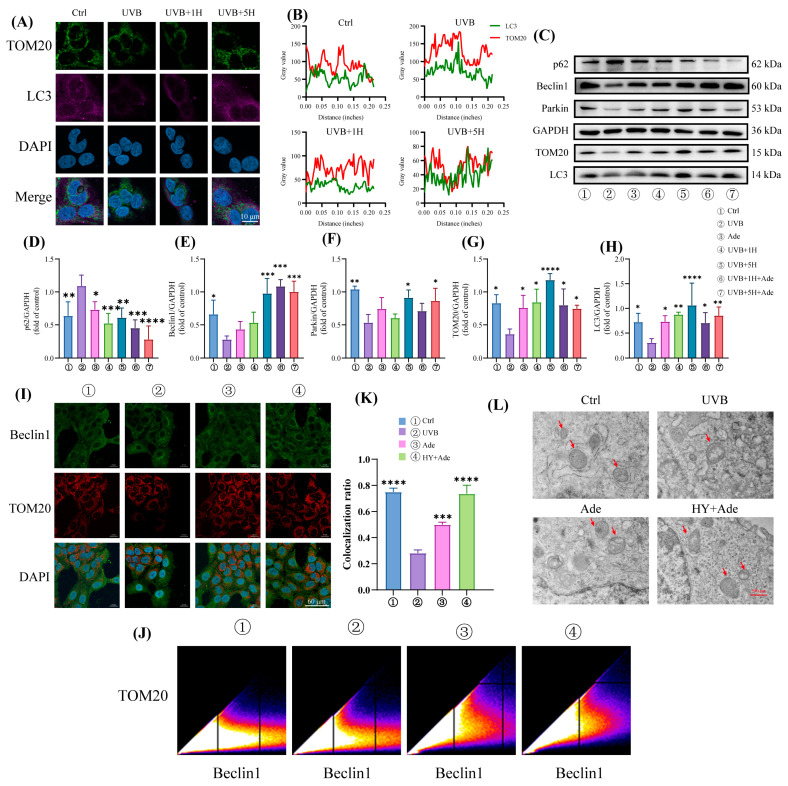
HY promotes mitochondrial autophagy. (**A**,**B**) Co-incubation staining with LC3 and TOM20 was observed by confocal microscopy (Scale bars: 10 μm). The cells were immunostained with TOM20 (green), LC3 (red) and Nuclei were stained with DAPI (blue). (**C**–**H**) Western blot was used to detect the expression of Beclin1, p62, LC3, Parkin, PINK1, and TOM20 in HaCaT cells and quantitatively analyze them. (**I**–**K**) Co-incubation staining with Beclin1 and TOM20 was observed by confocal microscopy (Scale bars: 60 μm). The cells were immunostained with TOM20 (red), Beclin1 (green) and Nuclei were stained with DAPI (blue). (**L**) Representative transmission electron microscopy images of the HaCaT cells in different treatment groups. Red arrows indicate mitochondria (Scale bars: 500 nm), highlighting the morphological changes across treatments. Data are presented as the mean ± SD (n = 3). * *p* < 0.05, ** *p* < 0.01, *** *p* < 0.001 and **** *p* < 0.0001. Ctrl (control group), UVB (the irradiated group), UVB 1H (1 μm of HY), UVB 5H (5 μm of HY).

HaCaT cells were subjected to immunofluorescence staining using antibodies against LC3 and TOM20. The results demonstrated that HY treatment increased the co-localization of LC3 and TOM20 compared to the UVB-treated group (Figure 4A,B). To further validate the induction of mitophagy, the mitophagy agonist Adezmapimod (Ade) was employed [31]. Western blot analysis revealed that Ade reversed the UVB-induced alterations in the expression of Beclin1, p62, LC3, Parkin, PINK1, and TOM20. Moreover, the combination of Ade and HY resulted in a more pronounced activation of mitophagy (Figure 4C–H). Immunofluorescence staining further confirmed that Ade enhanced the co-localization of Beclin1 and TOM20 in HaCaT cells, an effect that was significantly strengthened when Ade was used in combination with HY (Figure 4I–K). Additionally, transmission electron microscopy (TEM) was used to examine mitochondrial ultrastructure. The UVB group exhibited evident mitochondrial membrane damage, while autophagosomes were clearly observed in the HY-treated group. In conclusion, these results collectively indicate that HY promotes mitophagy, with enhanced efficacy observed when co-administered with Ade.

### 3.5. Network Pharmacology Predicts the Molecular Mechanisms of HY in the Prevention of Photoaging

**Figure 5:**
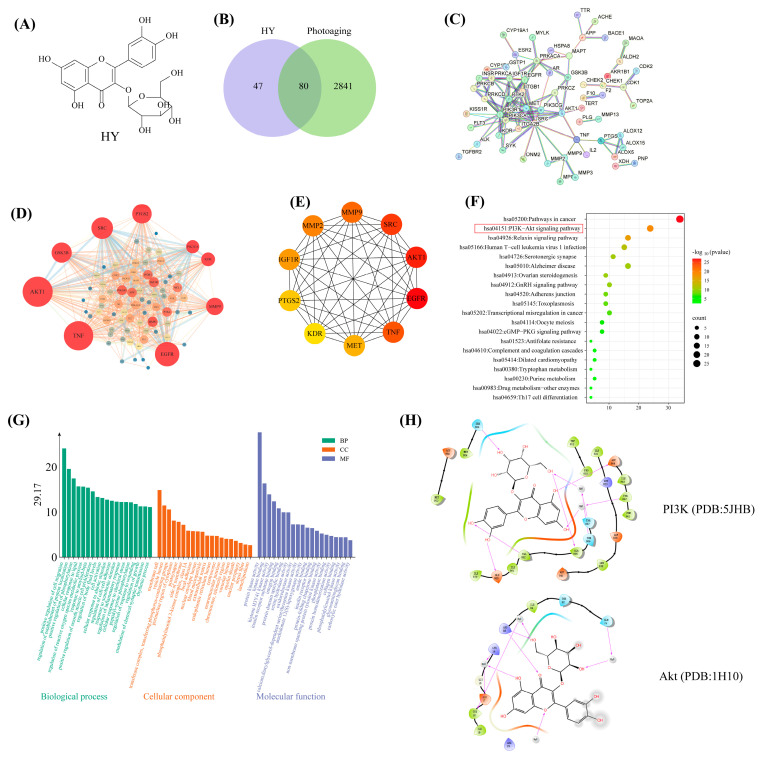
Molecular structure, potential targets, protein interaction network construction, and enrichment analysis of HY and photoaging. (**A**) The molecular structure of HY. (**B**) Venn diagram showing overlapping targets between HY and photoaging. (**C**) PPI networks based on common targets. (**D**,**E**) Screening and prediction of core targets. (**F**) KEGG pathway enrichment analysis top 20 terms. The size of the dots represents the number of enriched genes, and the color shade corresponds to the *p*-value. (**G**) Gene Ontology (GO) enrichment analysis top 60 terms. (**H**) Molecular docking of HY with PI3K and Akt.

Figure 5A illustrates the molecular structure of HY, a bioactive flavonoid compound. Potential protein targets of HY were systematically identified through database mining using SwissTargetPrediction (yielding 100 targets) and PharmMapper (yielding 35 targets). After removing duplicates, 127 unique potential target proteins were retained. Concurrently, photoaging-related targets were retrieved from the DisGeNET, GeneCards, OMIM, and DrugBank databases, resulting in 2921 non-redundant disease targets following deduplication. Venn diagram analysis revealed 80 common targets between HY and photoaging (Figure 5B). These overlapping targets were subsequently subjected to PPI network analysis. The PPI network was constructed using the STRING database (version 12.0) under strict parameters: organism limited to Homo sapiens, minimum interaction confidence score > 0.9, and exclusion of disconnected nodes (Figure 5C). The resulting network data in TSV format were imported into Cytoscape 3.10.3 for topological analysis and visualization (Figure 5D). Integrated network pharmacological analysis systematically identified Akt, SRC, and EGFR as key targets of HY (Figure 5E). Kyoto Encyclopedia of Genes and Genomes (KEGG) pathway enrichment analysis indicated that the differentially expressed genes are potentially involved in the PI3K/AKT signaling pathway (Figure 5F). Enrichment analysis of potential targets suggested that the anti-photoaging mechanism of HY primarily involves protein kinase activity and cell migration processes (Figure 5G). Given that PI3K is the most common upstream regulator of Akt and that Akt closely interacts with mTOR, the PI3K/Akt/mTOR signaling pathway—which plays crucial roles in cell survival, metastasis, metabolism, angiogenesis, and inflammatory recruitment—is likely central to HY’s activity [32]. Molecular docking results further demonstrated a strong binding affinity between HY and PI3K or Akt (Figure 5H). In summary, our integrated analysis predicts that HY exerts anti-photoaging effects primarily through modulation of the PI3K/Akt signaling pathway.

### 3.6. HY Activates Mitophagy by Inhibiting the PI3K/Akt/mTOR Signaling Pathway

**Figure 6:**
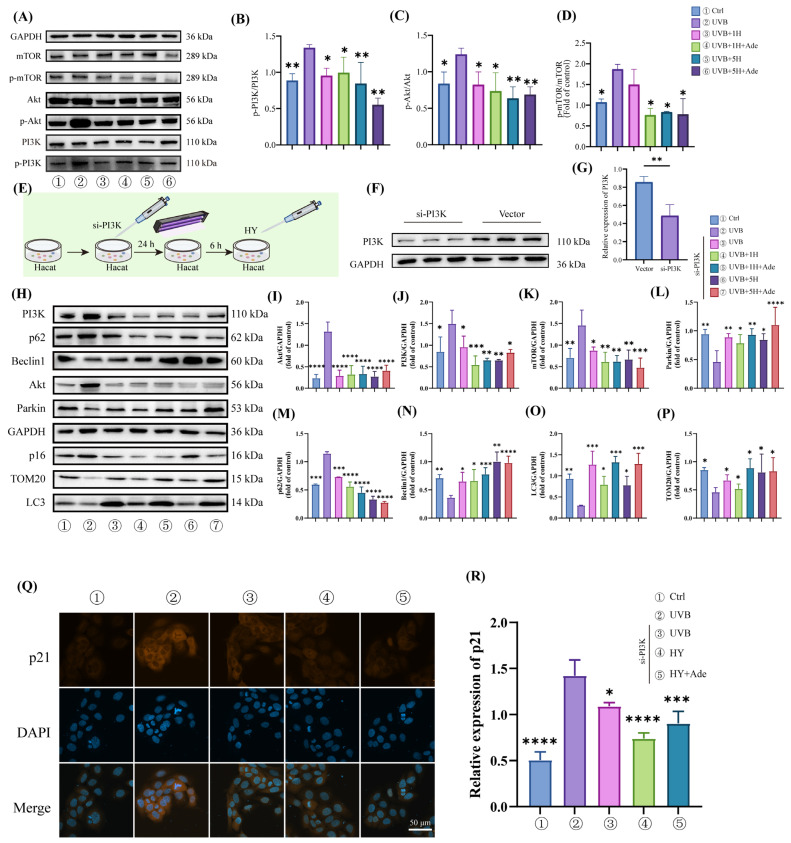
HY can activate mitochondrial autophagy by inhibiting PI3K/AKT/mTOR. (**A**–**D**) Western blot was used to detect PI3K, Akt, and mTOR, and their respective phosphorylation in HaCaT cells, and quantitatively analyzed. (**E**) Protocol for PI3K knockdown. (**F**,**G**) Western blot analysis of PI3K protein levels. (**H**–**P**) Western blot was used to detect the expression of PI3K, Akt, mTOR, Parkin, PINK1, Beclin1, p62, TOM20, and LC3 in HaCaT cells, and the results were quantitatively analyzed. (**Q**,**R**) Immunofluorescence detected the expression of p21 and quantified it (Scale bars: 50 μm). The cells were immunostained with p21 (red) and Nuclei were stained with DAPI (blue). Data are presented as the mean ± SD (n = 3). * *p* < 0.05, ** *p* < 0.01, *** *p* < 0.001 and **** *p* < 0.0001. Ctrl (control group), UVB (the irradiated group), UVB 1H (1 μm of HY), UVB 5H (5 μm of HY).

To further investigate whether HY regulates photoaging via the PI3K/Akt/mTOR signaling pathway, we examined the expression of phosphorylated PI3K (p-PI3K), Akt (p-Akt), and mTOR (p-mTOR) by Western blot. The results indicated that HY dose-dependently suppressed UVB-induced activation of the PI3K/Akt/mTOR pathway, and a synergistic effect was observed when HY was combined with Ade (Figure 6A–D). However, whether HY exerts its anti-photoaging effects by inhibiting the PI3K/Akt/mTOR pathway to activate mitophagy remained unclear. Therefore, we constructed PI3K-knockdown (si-PI3K) HaCaT cells using validated siRNA protocols (Figure 6E). Western blot analysis confirmed successful knockdown of PI3K protein expression (Figure 6F,G). Subsequent evaluation of mitophagy-related protein expression in si-PI3K cells revealed that Parkin, Beclin1, and PINK1 levels increased with rising HY concentration, exhibiting a clear concentration-dependent response. In contrast, TOM20 and LC3 levels did not show concentration dependence, and no significant synergistic effect was observed with Ade co-treatment (Figure 6H–P). These findings suggest the potential involvement of a negative feedback mechanism, which warrants further investigation. Immunofluorescence detection of p21 protein expression yielded consistent results (Figure 6Q,R). Taken together, these data indicate that HY likely activates mitophagy through inhibition of the PI3K/Akt/mTOR cascade to mitigate photoaging, though excessive suppression of this pathway may induce compensatory feedback responses.

### 3.7. HY Attenuates Cellular Senescence in HaCaT Cells by Inhibiting the miR-361-5p/PI3K/Akt/mTOR Signaling Pathway

In human skin, miRNAs serve critical regulatory functions in development, morphogenesis, and tissue homeostasis by modulating processes such as cell proliferation, differentiation, immune regulation, and wound healing [33]. By fine-tuning the expression of protein-coding genes, miRNAs act as subtle yet potent genetic regulators, making them promising candidates for maintaining skin health and counteracting premature aging [34,35]. Considering the pivotal role of the PI3K/Akt/mTOR signaling pathway in controlling cell proliferation, survival, and senescence [36], we hypothesized that its dysregulation represents a key mechanism underlying UVB-induced photoaging and that its activity may be modulated by upstream miRNAs. Therefore, this study aimed to identify specific miRNAs that regulate photoaging through the PI3K/Akt/mTOR pathway.

**Figure 7:**
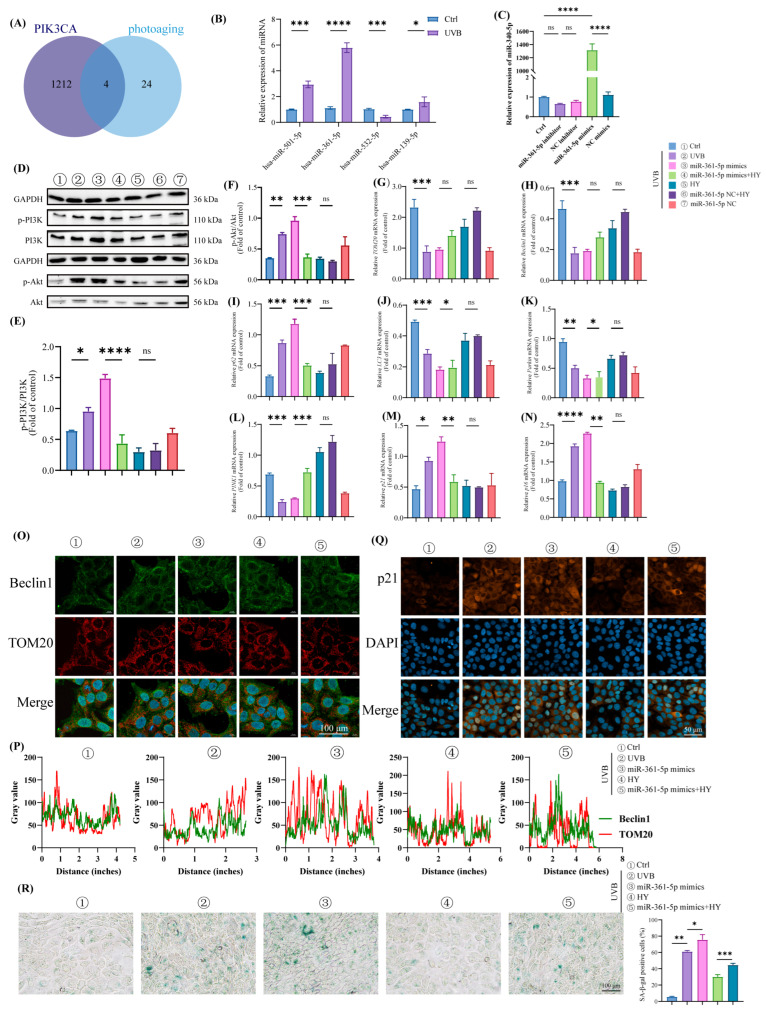
HY regulates the senescence of HaCaT cells by inhibiting the miR-361-5P/PI3K/Akt/mTOR signaling pathway. (**A**) Predicting the target gene of miR-130b-3p using bioinformatics tools. (**B**) RT-qPCR was used to detect the expression of has-miR-501-5p, has-miR-361-5p, has-miR-532-5p, and has-miR-139-5p. (**C**) Expression of miR-361-5p in THP-1 cells transfected with miR-361-5p mimics. (**D**–**F**) Western blot detected PI3K and Akt, along with their respective phosphorylation, in HaCaT cells and quantified them. (**G**–**N**) RT-qPCR detected *TOM20*, *Beclin1*, *p62*, *Parkin*, *PINK1*, *p21*, *p16* and LC3 in HaCaT cells and quantified them. (**O**,**P**) Co-incubation staining with Beclin1 and TOM20 was observed by confocal microscopy (Scale bars: 100 μm). The cells were immunostained with TOM20 (red), Beclin1 (green) and Nuclei were stained with DAPI (blue). (**Q**) Immunofluorescence detection of p21 expression (Scale bars: 50 μm). The cells were immunostained with p21 (red) and Nuclei were stained with DAPI (blue). (**R**) Representative image of SA-β-gal staining (Scale bars: 100 μm). Data are presented as the mean ± SD (n = 3). * *p* < 0.05, ** *p* < 0.01, *** *p* < 0.001 and **** *p* < 0.0001. Ctrl (control group), UVB (the irradiated group).

Based on our previous research, we identified 28 differentially expressed miRNAs (9 upregulated and 19 downregulated) in UVB-irradiated skin, with expression changes ranging from +1.7 to +8.4 (upregulation) and −1.9 to −25.0 (downregulation) [37]. Among these, miR-501-5p (+6.8) and miR-361-5p (+8.4) were the most significantly upregulated, while miR-23a (−25.0) and miR-323-3p (−7.7) were the most downregulated [37]. To identify miRNAs most likely to regulate the PI3K/Akt/mTOR pathway, we performed bioinformatic analysis using the miRDB (141 targets) and TargetScan (1190 targets) databases. Cross-referencing yielded four candidate miRNAs targeting PI3K with significant differential expression: miR-501-5p, miR-361-5p, miR-532-5p, and miR-139-5p (Figure 7A). RT-qPCR validation confirmed that miR-361-5p exhibited the most pronounced differential expression (Figure 7B), consistent with findings reported by Kraemer et al., further supporting the involvement of miRNAs in photoaging.

To investigate whether HY mediates its anti-photoaging effects via miR-361-5p in HaCaT cells, we constructed miR-361-5p mimics and confirmed their transfection efficiency (Figure 7C). Western blot analysis showed that miR-361-5p overexpression activated the PI3K/Akt pathway, whereas HY treatment inhibited its activation. Additionally, miR-361-5p mimic transfection suppressed mitophagy, an effect that was reversed by HY (Figure 7D–N). Confocal microscopy revealed reduced Beclin1 and TOM20 colocalization in miR-361-5p-overexpressing cells, which was significantly restored upon HY treatment (Figure 7O,P). We further evaluated the effect of HY on miR-361-5p-induced senescence using immunofluorescence and SA-β-gal staining. Compared to the UVB group, miR-361-5p-transfected cells showed elevated p21 expression, increased β-galactosidase activity, and a higher proportion of senescent cells. HY treatment counteracted these effects, reducing p21 levels, β-galactosidase activity, and senescence (Figure 7Q,R). Taken together, these results demonstrate that HY mitigates UVB-induced cellular senescence by downregulating miR-361-5p, thereby inhibiting the PI3K/Akt/mTOR pathway and promoting mitophagy.

### 3.8. HY Exhibits Potential in Inhibiting Skin Photoaging and Cellular Damage in Mice

**Figure 8:**
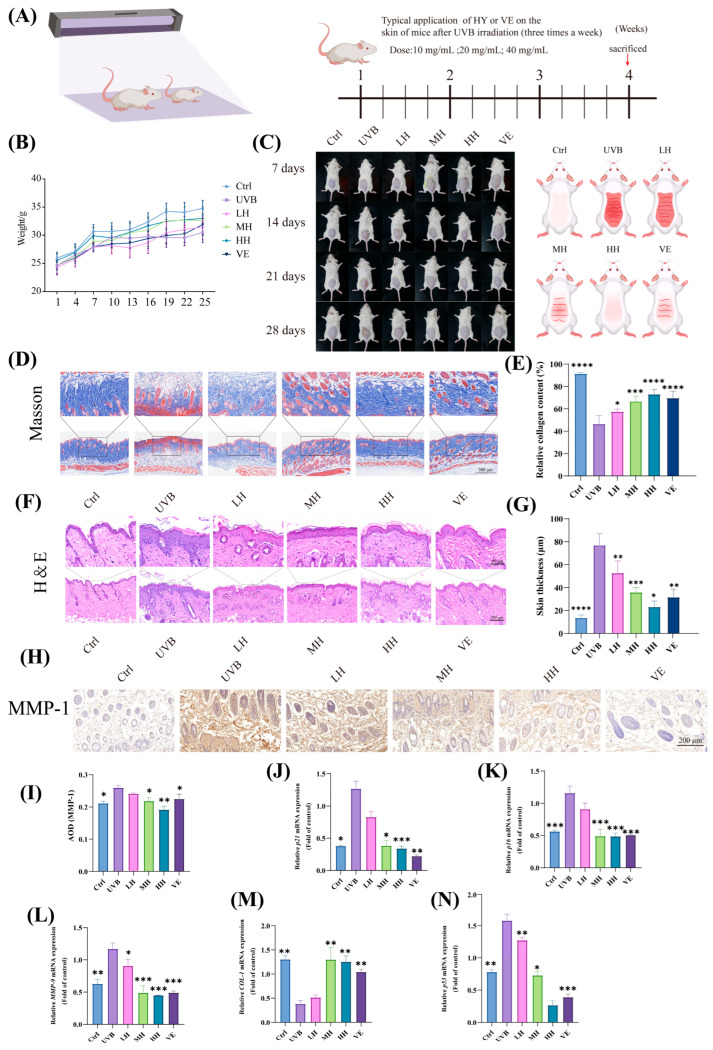
The effect of HY on UVB-induced photodamage and collagen loss in mice. (**A**) Schematic diagram of the experimental setup. (**B**) Representative photos of the dorsal skin of mice on days 1, 7, 14, 21, and 28 after the first treatment. Schematic representation of mice on day 28 after the first treatment. (**C**) Changes in body weight of mice every three days. (**D**,**E**) Masson staining and collagen content of the dorsal skin tissue of mice after 4 weeks of treatment, epidermal thickness (*n* = 8), full view scale bar = 300 μm. Scale bar in magnified local view = 400 μm. (**F**,**G**) H&E staining and epidermal thickness of the dorsal skin tissue of mice after 4 weeks of treatment (*n* = 8), full view scale bar = 200 μm. Scale bar in magnified local view = 80 μm. (**H**,**I**) Representative immunohistochemical (IHC) images of the dorsal skin of mice after 4 weeks of treatment, scale bar = 200 μm, n = 6. (**J**–**N**) After 4 weeks of treatment, the expression of *MMP-9*, *p16*, *p21*, *p53*, and *COL-1* was detected and quantified using RT-qPCR. Data are presented as the mean ± SD (n = 3). * *p* < 0.05, ** *p* < 0.01, *** *p* < 0.001, and **** *p* < 0.0001. Ctrl (control group), UVB (the irradiated group), LH (10 mg/mL of HY), MH (20 mg/mL of HY), HH (40 mg/mL of HY), and VE (40 mg/mL of VE).

Photoaging leads to significant deterioration of skin appearance, commonly manifested as wrinkles, laxity, erythema, and hyperpigmentation. The experimental design of the mouse photoaging model in this study is illustrated in Figure 8A. Treatment groups included LH, MH, HH, and a positive control VE group. Weekly photographic records were taken throughout the study. After 28 days, dorsal skin samples were collected to evaluate the effects of each treatment group on UVB-induced aging.

As shown in Figure 8B, the model group exhibited significant skin thickening, redness, and deep wrinkle formation, while all treatment groups showed varying degrees of improvement. The LH group still had slight redness and numerous wrinkles, with limited improvement; the MH group displayed mild sunburn with few wrinkles; both the HH and VE groups showed no obvious signs of photoaging, though fine wrinkles remained in the VE group. In terms of body weight, the model group showed suppressed weight gain, while the treatment groups exhibited a steady increasing trend (Figure 8C).

Histological analysis further revealed the protective effects of the treatments at the cellular level. Masson’s trichrome staining indicated a substantial loss of dermal collagen in the model group—approximately 45.12% reduction compared to the control. All treatment groups increased dermal collagen content to varying degrees, with the HH group showing a 51.31% increase compared to the model group (Figure 8D,E). H&E staining demonstrated hyperkeratosis, acanthosis, and inflammatory cell infiltration in the model group, which were alleviated to some extent in the LH, MH, and HH groups. The HH group showed the most significant effect, with an epidermal thickness of only 26.58 ± 2.13 μm, significantly lower than that of the model group (53.44 ± 6.73 μm) (Figure 8F,G).

Western blot analysis indicated decreased expression of *COL-1* and increased expression of *MMP-9, p21, p53,* and *p16* in the model group. These changes were markedly reversed in the HH group (Figure 8J–N). Immunohistochemical findings further confirmed these results, showing the highest MMP-1 expression in the UVB-induced model group and the lowest in the HH group (Figure 8H,I). In summary, UVB significantly degrades skin collagen and elastic fibers, leading to structural alterations and accelerated cellular senescence, while high-dose HY treatment effectively mitigates these changes, preserving skin structural and functional integrity.

## 4. Discussion

This study aimed to investigate the role of HY in skin photoaging and its underlying molecular mechanisms. Photoaging, primarily caused by chronic UVB exposure, is characterized by wrinkle formation, loss of skin elasticity, and degradation of the extracellular matrix [38,39]. Currently, effective therapeutic compounds against photoaging remain limited, highlighting the importance of identifying bioactive natural products with anti-photoaging potential [40].

This study unveils a novel mechanism through which HY alleviates skin photoaging via the miR-361-5p/PI3K/Akt/mTOR axis. Unlike many previously reported phytochemicals that primarily function through direct antioxidant mechanisms [3,41,42], HY acts on a more upstream target. It suppresses a specific microRNA, miR-361-5p, thereby derepressing the PI3K/Akt/mTOR pathway and initiating mitophagy. This mechanism intricately links intercellular communication (exosomal miRNA), key signaling pathways, and organelle quality control, offering a new paradigm for understanding how natural compounds precisely regulate cellular homeostasis. Our findings not only reinforce the central role of the PI3K/Akt/mTOR pathway in photoaging but, more importantly, reveal a previously unrecognized, miRNA-mediated layer of fine-tuning upstream of this pathway in response to natural compound intervention.

Although multiple miRNAs, such as miR-23a and miR-34a, have been reported to participate in photoaging, most exert their effects through antioxidant or anti-inflammatory pathways [43,44,45,46]. In alignment with previous work from our group, miR-361-5p appears to be unique in its ability to regulate mitochondrial function via the PI3K/Akt/mTOR pathway, revealing a new dimension in photoaging mechanisms. Moreover, unlike previously reported phytochemicals that act primarily through direct antioxidant mechanisms [3,41,42], HY specifically inhibits the PI3K/Akt/mTOR pathway, underscoring its distinctive mechanistic profile and potential therapeutic value.

In recent years, the incidence of skin disorders associated with UVB radiation has been increasing [38]. Mitochondrial dysfunction plays a central role in photoaging: UVB irradiation induces excessive mitochondrial reactive oxygen species (mtROS) production, leading to mitochondrial damage and impaired mitophagy, which in turn accelerates cellular senescence and SASP factor secretion, ultimately resulting in extracellular matrix degradation. HY is a natural compound with diverse pharmacological activities. Previous studies have reported that HY inhibits Akt phosphorylation and downregulates PI3K expression in liver cancer cells [47] and modulates autophagy via the PI3K/Akt and MAPK pathways in epilepsy models [48]. However, it remained unclear whether HY ameliorates photoaging in keratinocytes by regulating mitophagy.

The PI3K/Akt/mTOR pathway is a classic signaling cascade extensively involved in cell growth, metabolism, and aging [49,50]. In this study, potential targets of HY were screened using the SwissTargetPrediction and PharmMapper databases and intersected with known photoaging-related targets, suggesting PI3K/Akt/mTOR as a candidate pathway. Experimental results showed that UVB activates this pathway, while PI3K knockdown significantly suppressed p21 expression and enhanced mitophagy, indicating that inhibition of PI3K/Akt/m signaling alleviates photoaging phenotypes.

Exosomes act as paracrine mediators that can influence photoaging through the transfer of miRNAs [51]. Multiple studies have shown that UVB alters the miRNA expression profile in keratinocytes [52], with miR-124 among others being significantly upregulated in photoaged skin [53]. Through miRDB and TargetScan predictions followed by experimental validation, we identified miR-361-5p as an upstream regulator of the PI3K/Akt/mTOR pathway. Its expression increased after UVB irradiation, while HY treatment suppressed miR-361-5p, thereby inhibiting the PI3K/Akt/mTOR pathway and promoting mitophagy. Although bioinformatic analysis suggests that miR-361-5p may directly target PI3K, the precise binding mechanism requires further experimental validation.

While this study elucidates the photoprotective and anti-aging effects and mechanisms of HY through in vitro and in vivo studies, some limitations should be acknowledged. First, the research primarily focused on the short-term effects of HY on UVB-induced acute damage and aging models; its preventive efficacy against natural aging or long-term chronic photo-exposure requires further investigation. Second, the animal model used was mice, whose skin structure differs from that of humans. Therefore, the translational effect of HY in human applications needs to be confirmed by subsequent clinical trials. Finally, although we identified key pathways through molecular docking and experimental validation, the complete network of HY’s actions within the complex skin microenvironment may involve more intricate crosstalk, which represents a direction for future research.

## 5. Conclusions

In conclusion, our results demonstrate that HY effectively enhances HaCaT cell bioactivity, reduces SASP secretion, and downregulates the expression of senescence-associated proteins. Integrating our network pharmacology, molecular docking, and in vitro experimental evidence, we propose a potential model of action whereby HY may exert its anti-photoaging effects by modulating miR-361-5p, subsequently inhibiting the PI3K/Akt/mTOR pathway and activating mitophagy. These findings provide novel insights into the molecular mechanisms of photoaging.

Nevertheless, this study has several limitations. First, the proposed mechanistic pathway is primarily based on evidence from in vitro cell models and requires further validation in more complex in vivo animal models. Second, the direct interaction between HY and miR-361-5p has not been experimentally confirmed. Finally, the long-term in vivo safety and dermal absorption efficiency of HY require systematic evaluation. Despite these limitations, our study provides valuable preclinical evidence supporting HY as a candidate for photoaging intervention. Future work will focus on validating the proposed mechanism in vivo and further exploring the delivery strategies and safety profile of HY to lay the groundwork for its potential clinical translation.

## Figures and Tables

**Table 1:** Oligonucleotide primers used for real-time RT-PCR.

mRNA	Forward Sequence	Reverse Sequence
interleukin-1α (*IL-1α*)	AGATGCCTGAGATACCCAAAACC	CCAAGCACACCCAGTAGTCT
tumor necrosis factor-α (*TNF-α*)	CCTCTCTCTAATCAGCCCTCTG	GAGGACCTGGGAGTAGATGAG
*VEGF*	GAGATGTCCCTGGAAGAACACA	GAGTGGGATGGGTGATGTCAG
glyceraldehyde-3-phosphate dehydrogenase (*GAPDH*)	GGAGCGAGATCCCTCCAAAAT	GGCTGTTGTCATACTTCTCATGG
cyclin-dependent kinase inhibitor 1A (*p21*)	CCTGTCACTGTCTTGTACCCT	GCGTTTGGAGTGGTAGAAATCT
multiple tumor suppressor 1 (*p16*)	GGCACCAGAGGCAGTAACCAT	GCGCTACCTGATTCCAATTCG
tumor protein 53 (*p53*)	CAGCACATGACGGAGGTTGT	TCATCCAAATACTCCACACGC
translocase of outer mitochondrial membrane 20 (*TOM20*)	CTGCGTCGTGTTCCACTT	CTCCGCAACCTGACCATCT
*Beclin1*	GCTGGAAGATGCTCCTGACC	CAGTTGTTCTGGGAGGACCA
sequestosome 1 (*p62*)	GCACCCCAATGTGATCTGC	CGCTACACAAGTCGTAGTCTGG
*Parkin*	TGGATGGCTTCTCCGACTAC	AAGGTCCTGCCACTGCTC
PTEN-induced putative kinase 1 (*PINK1*)	CGCGGGAGTCAATGAGAAAA	GGCAGCAGAGGAAGGTGAAG
microtubule-associated protein light chain 3 (*LC3*)	AGCAGCATCCAACCAAAATC	CTGTGTCCGTTCACCAACAG
Collagen I (*COL-1*)	GAGGGCCAAGACGAAGACATC	CAGATCACGTCATCGCACAAC
matrix metalloproteinase-9 (*MMP-9*)	TGTACCGCTATGGTTACACTCG	GGCAGGGACAGTTGCTTCT

## Data Availability

The data are not publicly available due to privacy or ethical restrictions. The raw data supporting the conclusions of this article will be made available by the authors on request.

## Abbreviations

The following abbreviations are used in this manuscript:

UVB	ultraviolet radiation b
UV	ultraviolet
UVR	ultraviolet radiation
VEGFA	vascular endothelial growth factor A
VEGFR	vascular endothelial growth factor
FOXM1	forkhead box protein M1
PI3K	phosphatidyqinositol-3 kinase
HY	hyperoside
SASP	senescence-associated secretory phenotype
ROS	reactive oxygen species
HaCaT	human keratinocytes
AKT	protein kinase B
mTOR	mammalian target of rapamycin
miR	microRNA
MMP-1	matrix metalloproteinase-1
MMP-9	matrix metalloproteinase-9
COL-1	collagen I
IL-1α	interleukin-1α
TNF-α	tumor necrosis factor-α
VEGF	vascular endothelial growth factor
GAPDH	glyceraldehyde-3-phosphate dehydrogenase
p16	multiple tumor suppressor 1
p21	cyclin-dependent kinase inhibitor 1A
p53	tumor protein 53
p62	sequestosome 1
LC3	microtubule-associated protein light chain 3
PINK1	PTEN-induced putative kinase 1
TOM20	translocase of outer mitochondrial membrane 20
γ-H2AX	phosphorylated histone 2AX
DMEM	Dulbecco’s modified Eagle’s medium
FBS	fetal bovine serum
BCA	bicinchoninic acid
MTT	methylthiazolyldiphenyl-tetrazolium bromide
EdU	5-Ethynyl-2′-deoxyuridine
RT-qPCR	Quantitative Real-Time PCR
SEM	standard error of the mean
ANOVA	analysis of variance
SDS-PAGE	sodium dodecyl sulfate—polyacrylamide gel electrophoresis
PVDF	polyvinylidene fluoride
DCFH-DA	2′,7′-dichlorofluorescin diacetate
GO	Gene Ontology
KEGG	Kyoto Encyclopedia of Genes and Genomes
PPI	protein–protein interaction
ICR	Institute of Cancer Research
VE	vitamin E
ARRIVE	Animal Research: Reporting of In Vivo Experiments
IHC	immunohistochemical
DAB	3,3-*N*-diaminobenzidine tetrahydrochloride
LH	low-dose HY
MH	medium-dose HY
HH	high-dose HY
